# Evaluation of time profile reconstruction from complex two-color microarray designs

**DOI:** 10.1186/1471-2105-9-1

**Published:** 2008-01-03

**Authors:** Ana C Fierro, Raphael Thuret, Kristof Engelen, Gilles Bernot, Kathleen Marchal, Nicolas Pollet

**Affiliations:** 1 CNRS UMR 8080, Laboratoire Développement et Evolution, Bat 445, F-91405 Orsay, France; 2 Univ Paris Sud, F-91405 Orsay, France; 3 Programme d'Epigenomique – Genopole, Univ Evry, Tour Evry-2, Place des terrasses, 91000 Evry, France; 4 Dep Microbial and Molecular Sciences, K.U.Leuven, Kasteelpark Arenberg 20, 3001 Leuven, Belgium

## Abstract

**Background:**

As an alternative to the frequently used "reference design" for two-channel microarrays, other designs have been proposed. These designs have been shown to be more profitable from a theoretical point of view (more replicates of the conditions of interest for the same number of arrays). However, the interpretation of the measurements is less straightforward and a reconstruction method is needed to convert the observed ratios into the genuine profile of interest (e.g. a time profile). The potential advantages of using these alternative designs thus largely depend on the success of the profile reconstruction. Therefore, we compared to what extent different linear models agree with each other in reconstructing expression ratios and corresponding time profiles from a complex design.

**Results:**

On average the correlation between the estimated ratios was high, and all methods agreed with each other in predicting the same profile, especially for genes of which the expression profile showed a large variance across the different time points. Assessing the similarity in profile shape, it appears that, the more similar the underlying principles of the methods (model and input data), the more similar their results. Methods with a dye effect seemed more robust against array failure. The influence of a different normalization was not drastic and independent of the method used.

**Conclusion:**

Including a dye effect such as in the methods lmbr_dye, anovaFix and anovaMix compensates for residual dye related inconsistencies in the data and renders the results more robust against array failure. Including random effects requires more parameters to be estimated and is only advised when a design is used with a sufficient number of replicates. Because of this, we believe lmbr_dye, anovaFix and anovaMix are most appropriate for practical use.

## Background

Microarray experiments have become an important tool for biological studies, allowing the quantification of thousands of mRNA levels simultaneously. They are being customarily applied in current molecular biology practice.

In contrast to the Affymetrix based technology, for the two-channel microarray technology assays, mRNA extracted from two conditions is hybridised simultaneously on a given microarray. Which conditions to pair on the same array is a non trivial issue and relates to the choice of the "microarray design". The most intuitively interpretable and frequently used design is the "reference design" in which a single, fixed reference condition is chosen against which all conditions are compared. Alternatively, other designs have been proposed (e.g. a loop design). From a theoretical point of view, these alternative designs usually offer, at the same cost, more balanced measurements in the number of replicates per condition than a common reference design. They are thus, based on theoretical issues, potentially more profitable [1,2]. For instance, a loop design would outperform the common reference design when searching for differentially expressed genes [3]. However, the drawback of such alternative design is that the interpretation of the measurements becomes less straightforward. More complex analysis procedures are needed to reconstruct the factor of interest (genes being differentially expressed between two particular conditions, a time profile, etc.), so that the practical usefulness of a design depends mainly on how well analysis methods are able to retrieve this factor of interest from the data. Such analysis would require removing systematic biases from the raw data by the appropriate normalization steps and combining replicate values to reconstruct the factor of interest.

When focusing on profiling the changes in gene expression over time, the factor of interest is the time profile [1,2]. For such time series experiments, the "reference design", where, for instance, time point zero is chosen as the common reference has a straightforward interpretation: for each array, the genes' mean ratio between replicates readily represents the changes in expression of that gene relative to the first time point. However, when using an alternative design, such as an interwoven design, mean ratios represent the mutual comparison between distinct (sometimes consecutive) time points. A reconstruction procedure is needed to obtain the time profile from the observed ratios [3-5].

Several profile reconstruction methods are available for complex designs. They all rely on linear models, and for the purpose of this study, we subdivided them in "gene-specific" and "two-stage" methods. Gene-specific profile reconstruction methods apply a linear model to each gene separately. The underlying linear model is usually only designed for reconstructing a specific gene profile from a complex design, but not for normalizing the data. As a result, normalized log-ratios are used as input to these methods (see 'Methods'). Examples of these methods are described by Vinciotti, *et al*. (2005) [3] and Smyth, *et al*. (2004) (Limma) [4]. Two-stage profile reconstruction methods on the other hand, first apply a single linear model to all data simultaneously, i.e. the model is fitted to the dataset as a whole. These models use the separate log-intensity values for each channel, as spot effects are explicitly incorporated. They return normalized absolute expression levels for each channel separately, which can then be used to reconstruct the required time profile by a second-stage gene-specific model. An example of such two-stage method is implemented in the MAANOVA package [6].

So far, comparative studies focused on the ability of different methods to reconstruct "genes being differentially expressed" from different two-color array based designs [7-9] or the ratio estimation between two particular conditions [5]. In this study, we aimed at performing a comparative study focusing on the time profile as the factor of interest to be reconstructed from the data.

We compared to what extent five existing profile reconstruction methods (lmbr, lmbr_dye, limmaQual, anovaFix, and anovaMix; see 'Methods' for details) were able to reconstruct similar profiles from data obtained by two channel microarrays using either a loop design or an interwoven design. We assessed similarities between the methods, their sensitivity towards using alternative normalizations and their robustness against array failure. A spike-in experiment was used to assess the accuracy of the ratio estimates.

## Results

### Assessing the influence of the used methodology on the profile reconstruction

We compared to what extent the different methods agreed with each other in 1) estimating the changes in gene expression relative to the first time point (i.e. the log-ratios of each single time point and the first time point) and 2) in estimating the overall gene-specific profile shapes. Results were evaluated using two test sets, each of which represents a different complex design.

**Figure 1:**
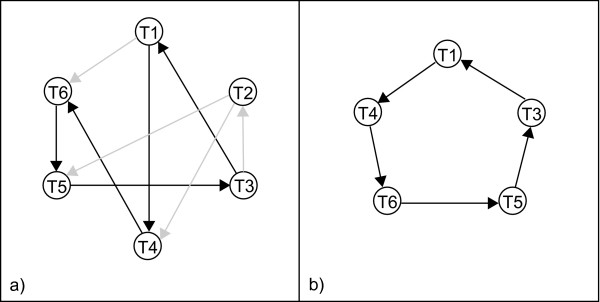
**Experimental microarray designs used in this study**. Circles represent samples or time points, and arrows represent a direct hybridization between two samples. The arrows point from the time point labeled with Cy3 to the time point labeled with Cy5. (a) Interwoven design (first dataset). Grey arrows were removed to generate a single loop design (see (b)). (b) Loop design (second dataset).

The first dataset was a time series experiment consisting of 6 time points measured on 9 arrays using an interwoven design (Figure 1a). This design resulted in three replicate measurements for each time point, with alternating dyes. As a second test, a smaller loop design was derived from the previous dataset by picking the combination of five arrays that connect five time points in a single loop (Figure 1b). A balanced loop is obtained with two replicates per condition, for which each condition is labeled once with the red and once with the green dye (see 'Methods')

The balance with respect to the dyes (present in the loop design) ensures that the effect of interest is not confounded with other sources of variation. In this study, the effect of interest corresponds to the time profile. The replication (as present in the interwoven design) improves the precision of the estimates and provides the essential degrees of freedom for error estimation [2]. Moreover, the interwoven design not only has more replicates, but also increases the possible paths to join any two conditions in the design. As they have different characteristics, using both datasets allows us to assess the reconstruction process under two different settings, while the RNA preparations for both designs are the same.

#### Effect of profile reconstruction methods on the ratio estimates

We first assessed to what extent the different methods agreed with each other in estimating similar log-ratios for each single gene at each single time point. To this end, we calculated the overall correlation per time point between the gene expression ratios estimated by each pair of two different methods. Table 1 gives the results for all mutual comparisons between the methods tested for the loop design. Irrespective of which two methods were compared, the correlation between the estimated ratios was high on average, ranging from 0.94 to 0.98 (Table 1, mean column). Moreover, this high average correlation is due to a high correlation of all individual ratios throughout the complete ratio range (see Additional file 1), with only a few outliers (genes for which a rather different ratio estimate was obtained, depending on the method used). Note that for the loop design, there was no difference between the results of lmbr and lmbr_dye due to the balanced nature of this design (see 'Methods' section).

**Table 1:** Pairwise correlation between ratios estimates for the loop design

Method 1	Method 2	T3/T1	T4/T1	T5/T1	T6/T1	Mean
lmbr/lmbr_dye	limmaQual	0.9966	0.9998	0.9648	0.9909	0.9880
lmbr/lmbr_dye	anovaFix	0.9899	0.9913	0.9721	0.9856	0.9848
lmbr/lmbr_dye	anovaMix	0.9829	0.9751	0.9420	0.9549	0.9637
limmaQual	anovaFix	0.9889	0.9918	0.9298	0.9775	0.9720
limmaQual	anovaMix	0.9810	0.9758	0.8950	0.9467	0.9496
anovaFix	anovaMix	0.9936	0.9847	0.9726	0.9694	0.9801

Correlation between ratios estimated by each pair of applied methods (column 1 and column 2) for a loop design. Each ratio corresponds to a time dependent change in expression as compared to the first time point (T1). The last column corresponds to the mean correlation of the 4 estimates. Since the loop design is balanced with respect to the dyes, the results for lmbr and lmbr_dye were the same (see 'Methods' section), which is why they are not treated differently.

For this loop design the ratio estimates T3/T1 or T4/T1 obtained by each of the different methods are on overall more correlated than estimates of respectively T5/T1 and T6/T1. As can be expected, direct estimates, i.e. estimates of a ratio for which the measurements were assessed on the same array (see Figure 1b: ratios T3/T1 and T4/T1) are more consistent than indirect estimates, i.e. the measurements used to obtain the estimates were assessed on different arrays (see Figure 1b: ratios T5/T1 and T6/T1). A similar observation was already made by Kerr and Churchill (2001) [2], and Yang and Speed (2002) [10]. For a loop design, both the ANOVA (two-stage) [2] and the gene-specific methods [10], have trouble estimating ratios between conditions not measured on the same array (indirect estimates). The larger the loops (the longer the paths) between indirectly measured pairs of conditions, the less precise estimates will be.

For the interwoven design, the correlation between ratio estimates, obtained by any pair of two different methods was even higher, with values ranging from 0.95 to 0.99 (see Additional file 2). For this unbalanced design, the ratio estimates for the lmbr_dye and the lmbr methods were no longer exactly the same. The difference in consistency between direct and indirect ratio estimates was not obviously visible for this design.

#### Effect of profile reconstruction methods on the profile shape

A high average correlation between the ratio estimates obtained by the different methods at each single time point is a first valuable assessment. However, it is biologically more important that gene-specific profiles reconstructed by the different methods exhibit the same tendency over time. Therefore, we also compared to what extent profile shapes estimated by each of the methods differed from each other. This was done by computing the mean similarity between profile estimates obtained by any combination of two methods (Table 2).

**Table 2:** Mean similarity between profiles for both the interwoven and the loop design

	Interwoven design				Loop design		
	lmbr	lmbr_dye	limmaQual	anovaFix	lmbr/lmbr_dye	limmaQual	anovaFix

lmbr_dye	0.9477				1.0000		
limmaQual	0.9940	0.9359			0.9844		
anovaFix	0.9321	0.9572	0.9157		0.9514	0.9252	
anovaMix	0.9138	0.9373	0.8989	0.9767	0.9186	0.8934	0.9611

Values in the table correspond to the similarity between any two methods, expressed as the mean profile similarity of the genes. Since the loop design is balanced with respect to the dyes, the results for lmbr and lmbr_dye were the same (see 'Methods' section), which is why they are not treated differently. No filtering applied and similarity is assessed for all 2999 profile estimates.

**Figure 2:**
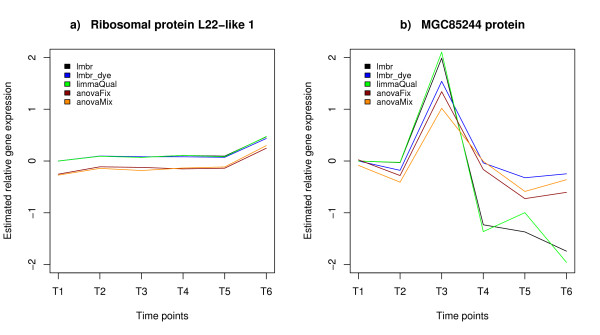
**Examples of reconstructed profiles for two representative genes from the interwoven design**. For the gene-specific methods (based on log-ratios estimate) the ratios are expressed relative to T1 and the ratio T1/T1 is set to zero. For the two-stage (ANOVA) methods, the estimated VG effect (gene-variety) is plotted. (a) Estimated profiles for the Ribosomal like gene under the interwoven design. (b) Reconstructed time profile for the gene MGC85244 under the interwoven design.

Figure 2 shows a few illustrative examples of profiles estimated by the different methods. For the ribosomal gene "L22" (Figure 2a), irrespective of the method, highly similar profiles were obtained. However, for the MGC85244 gene (Figure 2c), the observed degree of similarity between profiles derived by each of the different methods is much lower, especially for the last two time points.

Table 2 summarizes the results of the profile comparison expressed as average profile similarities across all genes. The similarity was computed with the cosine similarity measure after mean centering the profiles (see 'Methods'). It ranges from -1 (anti-correlation) to 1 (perfect correlation), 0 being no correlation. Also here, the overall correlation between different methods was not drastically different. From this table, it appears that the more similar the underlying principles of the used methods (both the model and the input data) are, the more correlated their results. Indeed, correlations between profiles estimated by either limmaQual and lmbr (both gene-specific models without dye effect), or anovaMix and anovaFix (both two-stage models) are high. The most divergent correlations are observed when comparing a gene-specific method (more specifically lmbr, or limmaQual) with a two-stage method (anovaFix or anovaMix). When using lmbr_dye on the interwoven design, it behaves somewhere in between: although it is a single gene model, it includes a dye effect just like the two-stage models. This does not apply for the loop design due to its dye-balance (lmbr and lmbr_dye give the same results for balanced designs; see 'Methods').

Differences in the input data (log-ratio versus log-expression values) and alterations in the underlying model (including a dye or random effect) are confounded in affecting the final result. Therefore, in order to assess in more detail the specific effect of including either a dye or a random effect in the model, we compared results between methods that share the same input data.

To assess the influence of including a dye effect on profile estimation, we compared the results of the gene-specific methods (see Table 2, the first two rows). Including a dye effect (present in lmbr_dye but not in limmaQual and lmbr) has a strong effect under the unbalanced interwoven design (seen as decrease in correlation between lmbr_dye and the other single gene methods). For the loop design this effect is non-existent because of the loop design's balance with respect to the dyes (see 'Methods').

The mere impact of including a random effect in the model can be assessed by comparing results of anovaFix and anovaMix. Indeed, they both contain the same input data, the same normalization procedure, and the same model except for the random effect. Seemingly, inclusion of the random effect has a higher influence on the loop design than on the interwoven design.

Usually in a microarray experiment, an important proportion of the genes does not change its expression significantly under the conditions tested (global normalization assumption), exhibiting a "flat" profile. We wondered whether removing such flat genes, with a noisy profile would affect the similarity in profile estimation between the different methods. Indeed, because the cosine similarity with centering only measures the similarity in profile shape, regardless of its absolute expression level, the higher level of similarity we observe between the methods might be due to a high level of random correlation between the "flat" profiles. Therefore, we applied a filtering procedure by removing those genes for which the profile variance over the different time points was lower than a certain threshold (a range of threshold values going from 0.2–0.4 was tested). The similarity was assessed for any pair of profile estimates corresponding to the same gene if at least one of the two profiles passed the filter threshold (Table 3 for the variance threshold of 0.4, results for the other thresholds can be found in the supplementary information, see Additional file 3).

**Table 3:** Mean similarity between profiles after filtering for both the interwoven and the loop design

	Interwoven design				Loop design		
	lmbr	lmbr_dye	limmaQual	anovaFix	lmbr/lmbr_dye	limmaQual	anovaFix

lmbr_dye	0.9834				1.0000		
limmaQual	0.9980	0.9799			0.9920		
anovaFix	0.9800	0.9948	0.9755		0.9961	0.9851	
anovaMix	0.9748	0.9905	0.9701	0.9957	0.9866	0.9728	0.9905

Mean profile similarity using a filtering threshold of 0.4 on all profiles estimated by each of the methods. A pairwise similarity comparison is made for all profile pairs (corresponding to the same gene) estimated by each of the two methods compared, for which at least one profile is above the filtering threshold.

Overall, the results obtained with each of the different variance thresholds confirmed the observations of Table 2: 1) the more similar the models and input data, the more similar the methods behaved (two-stage methods differed most from limmaQual followed by lmbr in estimating the gene profiles), 2) including a dye effect has a pronounced effect in an interwoven design (in a loop design there is no distinction due to the balance with respect to the dyes; see 'Methods'), 3) including a random effect has most influence on the loop design. In addition, it seems that, the more flat profiles are filtered from the dataset, the more similar the results obtained by each of the different methods become.

### The effect of array failure on the profile reconstruction

In practice, when performing a microarray experiment some arrays might fail with their measurements falling below standard quality. When these bad measurements are removed from the analysis, the complete design and the results inferred from it will be affected. Here we evaluated this issue experimentally by simulating array defects. In a first experiment, the interwoven design (dataset 1) was considered as the original design without failure. We tested 9 different possible situations of failure, by each time removing a single array from the design, resulting in 9 reduced datasets. The same test was performed with the loop design (dataset 2).

We compared for each of the different profile reconstruction methods the mean similarity between the ratios obtained either with the full dataset or with each of the reduced datasets (9 comparisons). Table 4 summarizes the results for the interwoven design, and Table 5 for the loop design.

**Table 4:** Assessing the effect of array failure on estimated ratios for the interwoven design

Array removed	lmbr	lmbr_dye	limmaQual	anovaFix	anovaMix	Conditions affected
1	0.9615	0.9812	0.9623	0.9824	0.9784	T2/T3
2	0.8905	0.9277	0.8768	0.9317	0.9431	T1/T3
3	0.9606	0.8790	0.9521	0.8951	0.8943	T3/T5
4	0.9080	0.9353	0.8821	0.9478	0.9539	T4/T1
5	0.9601	0.9632	0.9505	0.9569	0.9592	T6/T1
6	0.9773	0.9863	0.9742	0.9852	0.9847	T5/T2
7	0.9238	0.9585	0.9317	0.9549	0.9662	T5/T6
8	0.9615	0.9599	0.9615	0.9617	0.9690	T2/T4
9	0.9816	0.9859	0.9613	0.9836	0.9845	T4/T6

Mean	0.9472	0.9530	0.9392	0.9555	0.9592	

The different methods for which the influence of array failure was assessed are represented in the columns. Each row shows the mean correlation between the corresponding estimated ratios from the complete design and those obtained from a defect design (where one array was removed compared to the complete design). Mean: shows the overall mean correlation for a given method.

**Table 5:** Assessing the effect of array failure on estimated ratios for the loop design

Array removed	lmbr	anovaFix	anovaMix	Conditions affected
1	0.5964	0.6461	0.5312	T1/T3
2	0.7161	0.7545	0.6559	T3/T5
3	0.6713	0.8883	0.7606	T5/T6
4	0.5697	0.7883	0.6637	T6/T4
5	0.4359	0.6042	0.5534	T4/T1

Mean	0.5979	0.7363	0.6330	

The different methods for which the influence of array failure was assessed are represented in the columns. Each row shows the mean correlation between the corresponding estimated ratios from the complete design and those obtained from a defect design (where one array was removed compared to the complete design). Mean: shows the overall mean correlation for a given method. Lmbr_dye and limmaQual were not evaluated as in this particular case they lose their main differing characteristic compared to lmbr.

For the interwoven design (Table 4), it appears that in general removing one array from the original design did not really affect the ratio reconstruction. For all methods, ratio estimates tend to be more affected when an array measuring the reference time point was removed (T1) (Table 4). Overall the two-stage methods, and in particular anovaMix, seemed most robust against array failure, while limmaQual was most sensitive (Table 4). Methods including a dye effect were more robust against array failure. Similar results were obtained when the effect of array failure was assessed on the similarity in profiles (see Additional file 4).

For the loop design, the situation was quite different (Table 5). Note that here, the lmbr_dye and limmaQual methods were not used for profile reconstruction as the reduced datasets did not contain sufficient information for estimating all the model parameters. For both lmbr_dye and limmaQual, the linear models lose their main differing characteristics compared to lmbr (see 'Methods' section). For all remaining methods removing one array from the design affected the results considerably more than was the case for the interwoven design. Two-stage methods were the most robust, but in this design anovaMix performs slightly worse than anovaFix. The lmbr method turned out to be very sensitive to array failure, giving a mean profile similarity around 0.2, indicating no correlation between profiles estimated with and without array failure (see Additional file 5).

Note that overall, all methods seem to be more robust to array failure under the interwoven design than under the loop design. This is to be expected as the latter design contains more replicates.

### Consistency of the methods under different normalization procedures

In the previous section we compared profiles and ratio estimates obtained by the different methods after applying default normalization steps. However, other normalization strategies are possible, and could potentially affect the outcome. To assess the influence of using alternative normalization procedures, we compared profiles reconstructed from data normalized with 1) print tip Loess without additional normalization step (the default setting for anovaMix and anovaFix as used throughout this paper), 2) print tip Loess with a scale-based normalization between arrays [11], and 3) print tip Loess with a quantile-based between array normalization [12,13] (the default normalization for lmbr, lmbr_dye, and limmaQual as used throughout this paper).

Table 6 shows, for each of the different methods, the mean similarity between reconstructed profiles derived from differently normalized datasets. Overall, the influence of the normalization was not drastic. More importantly, the influence of the additional normalization steps seemed independent of the method used (similar influences were observed for all methods). When assessing the similarity in ratio estimates instead of profile estimates, similar results were obtained (data not shown).

**Table 6:** Effect of additional normalization procedures on estimating gene profiles from both the interwoven design and the loop design

	Interwoven design				
Normalization methods	lmbr	lmbr_dye	limmaQual	anovaFix	anovaMix

none/quantile	0.9602	0.9576	0.9599	0.9605	0.9584
none/scale	0.9900	0.9881	0.9914	0.9867	0.9848
scale/quantile	0.9540	0.9531	0.9557	0.9547	0.9566

	Loop design				

	lmbr	lmbr_dye	limmaQual	anovaFix	anovaMix

none/quantile	0.9520	0.9520	0.9534	0.9601	0.9481
none/scale	0.9822	0.9822	0.9793	0.9835	0.9778
scale/quantile	0.9355	0.9355	0.9361	0.9433	0.9370

Similarities between profiles were assessed using the mean cosine similarity measure. Rows indicate the different normalization procedures. Columns indicate the different models used to reconstruct the profiles.

### Accuracy of estimation

So far we only assessed to what extent changes in the used methodologies or normalization steps affected the inferred profiles. This, however, does not give any information on the accuracy of the methods, i.e., which of these methods is able to best approximate the true time profiles. Assessing the accuracy is almost impossible as usually the true underlying time profile is not known. However, datasets that contain external controls (spikes) could prove useful in this regard. Spikes are added to the hybridisation solution in known quantities, so that we have a clear view of their actual profile. In the following analysis, we used a publicly available spike-in experiment in attempt to assess the accuracy of each of the profile reconstruction methods [14]. For the technical details of this dataset we refer to 'Methods' and Table 7.

**Table 7:** Concentration (copies per cell) of the control clones spiked

Spike No.	Spike Mix 1	Spike Mix 2	Spike Mix 3	Spike Mix 4	Spike Mix 5	Spike Mix 6	Spike Mix 7	Reference Mix
1, 2	10,000	0	0.1	1	10	100	1,000	100
3, 4	1,000	10,000	0	0.1	1	10	100	100
5, 6	100	1,000	10,000	0	0.1	1	10	100
7, 8	10	100	1,000	10,000	0	0.1	1	100
9, 10	1	10	100	1,000	10,000	0	0.1	100
11, 11a	0.1	1	10	100	1,000	10,000	0	100
12, 13	0	0.1	1	10	100	1,000	10,000	100

From the total of 14 arrays, 7 were hybridized with the respective spike mixes labeled in Cy5 against the reference mix labeled in Cy3. The remaining 7 arrays were hybridized with the spike mixes labeled in Cy3 against the reference mix labeled in Cy5. Spike 11a was removed from analysis due to quality issues (Allemeersch et al., 2005 [17]).

**Figure 3:**
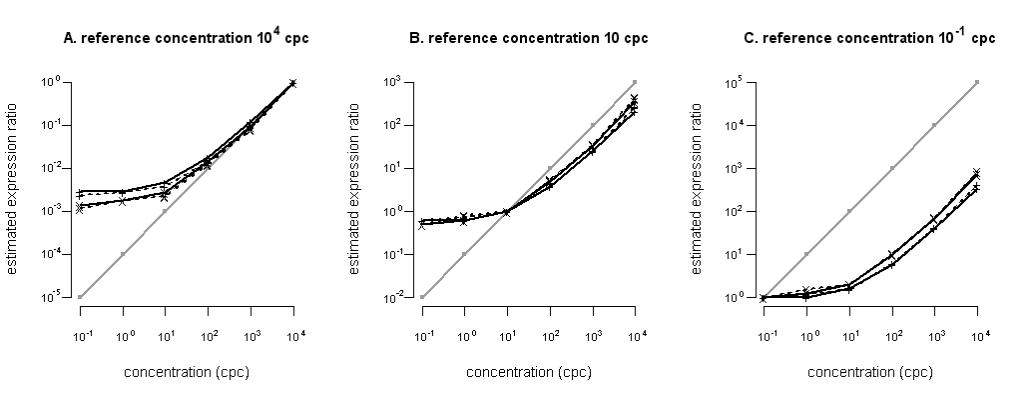
**Spike-in expression ratio estimates**. Reconstructed expression ratio estimates of spikes 7 (+ markers) and 8 (x markers) are plotted for lmbr/lmbr_dye/limmaQual (solid line), anovaFix (dotted line), and anovaMix (dashed line). Concentrations (cpc; copies per cell) of 10^4 ^cpc (panel A), 10 cpc (panel B), and 10^-1 ^cpc (panel C) were used as reference points. Estimated ratios were sorted from low to high concentrations. The solid grey line (o-markers) corresponds to the expected ratios for the known concentrations.

As lmbr and lmbr_dye and limmaQual gave exactly the same results using this balanced design, we further assessed to what extent lmbr, anovaFix and anovaMix agreed with each other. Fig. 3 shows the effect of using different spike concentrations as reference points for ratio estimation. Panels A through C reflect decreasing reference concentrations. The choice of reference has little effect on the shape of the profile (as indicated by consistent relationships between the different estimates). However, Fig. 3 illustrates that 1) lower reference concentrations (intensities) introduce a bias in the profile (true ratio's are consistently underestimated), 2) irrespective of the concentration of the reference ratio's derived for the lower expression values of the test are nearly identical, and thus uninformative. Both observations can be attributed to the lower saturation characteristics of microarray data (low concentrations do not generate signals that are distinguishable from the background). Although not as complex as the previously used loop or interwoven designs, the spiked-in design illustrates that this lower saturation effect, an inherent property of microarray data, can distort estimated profiles: interpretation of ratios with lower signals for test or reference should be done with care.

## Discussion

In this study, we evaluated the performance of five methods based on linear models in estimating gene expression ratios and reconstructing time profiles from complex microarray experiments. From a theoretical viewpoint, two major differences can be distinguished between the methods selected for this study: 1) differences related to alterations in the input data: the selected two-stage methods make use of the log-intensity values while the gene-specific methods use log-ratios, 2) differences related to the model characteristics: some of the models include an explicit dye effect (lmbr_dye, anovaFix and anovaMix) or an explicit random effect (anovaMix).

Although Kerr [5] assumed that observed differences in estimates obtained by different models are due to the differences in model characteristics, rather than to the input data, we cannot clearly make this distinction. Indeed, the way the error-term is modeled influences the statistical inference and hence the use of log-intensities or log-ratios does cause a difference between models [5]. However, when focusing on results obtained between methods with similar input data, we can assess, to some extent, the effect of different model specificities. In the following sections, some of these effects are discussed more in detail.

### The inclusion of the dye effect

In general we observed that, gene-specific methods without dye effects, and two-stage models with dye effect behaved more similar with each other than when they were compared among each other. Lmbr_dye (a gene-specific model with dye effect) is situated somewhere in between when the design is unbalanced with respect to the dyes. Indeed, the gene-specific models lmbr and limmaQual contain a combination of log-ratios plus an error term. However, when adding a dye effect to these models as is the case of lmbr_dye, the formulations and estimations converge with those of the two-stage ANOVA models for unbalanced designs.

Originally, Vinciotti, *et al.* (2005) [3] and Wit, *et al.* (2005) [15] added the dye effect for purposes of data normalization when one is working with non-normalized data. From our results, we also noted a practical advantage of including a dye effect even with normalized data. The fact that adding a dye effect showed pronounced differences for a dye-unbalanced design indicates that, despite the data being normalized, there are still dye-related inconsistencies in the data that might -partially- be compensated for by including a dye effect. Moreover, models with dye effects seemed more robust in estimating log-ratios from a design disturbed by array failure. Therefore, when working with unbalanced designs, it is advisable to include a dye effect, not only for the two-stage ANOVA models, as was also suggested by Wolfinger (2001) [16], Kerr (2003) [5], and Kerr and Churchill (2001) [2], but also for gene-specific models based on log-ratios.

### Mixed models versus Fixed models

Several studies advise the users to model the spot-gene or array-gene effects as random variables [9,16]. We observed that under the loop design (with 5 arrays), profiles estimated by anovaMix and anovaFix diverged. We also noticed that, for the loop design anovaMix had a lower capacity than anovaFix to handle array failures. For the interwoven design with 9 arrays these effects were less pronounced. The loop design used in our study does not contain a sufficient number of replicates to allow for reliable estimation of the spot-gene effect when using a mixed ANOVA model. As a result, ratios and time profiles estimated by anovaMix than anovaFix are less reliable for an experiment with few replicates

### The effect of using alternative normalization steps on the methods' performance

We tested the influence of using additional normalization steps. Differently normalized data give different results, but the effects were not dramatic. Moreover, they had the same influence on all methods, indicating that all methods were equally sensitive to changes in the normalization.

### Accuracy of estimated ratios

Based on spike-in experiments for two-channel microarrays, we could also assess to what extent the estimated ratios approximated the true ratios (i.e., the accuracy of the estimated ratios). We observed that all five tested linear methods generated biased estimations, consistently overestimating changes in expression relative to a reference with low mRNA-concentration. These results were independent of the method used (gene-specific or two-stage) or of the number of effects included the model.

## Conclusion

On average the correlation between the estimated ratios was high, and all methods more or less agreed with each other in predicting the same profile. The similarity in profile estimation between the different methods improved with an increasing variance of the expression profiles.

We observed that when dealing with unbalanced designs, including a dye effect, such as in the methods lmbr_dye, anovaFix and anovaMix, seems to compensate for residual dye related inconsistencies in the data (despite an earlier normalization step). Adding a dye effect also renders the results more robust against array failure. Including random effects requires more parameters to be estimated and is only advised when a design is used with a sufficient number of replicates.

Conclusively, because of their robustness against imbalances in the design and array failure, we believe lmbr_dye, anovaFix and anovaMix are most appropriate for practical use (given a sufficient number of replicates in case of the latter).

## Methods

### Microarray data

The first dataset used in this study was a temporal *Xenopus tropicalis *expression profiling experiment. The array used consisted of 2999 oligos of 50 mers, corresponding to 2898 unique *X. tropicalis *gene sequences and negative control spots (*Arabidopsis thaliana *probes, blanks and empty buffer controls). Each oligo was spotted in duplicate on each array in two separated grids. On each grid, oligonucleotides were spotted in 16 blocks of 14 × 14 spots. Pairs of duplicated oligo's on the two grids of the same gene sequence were treated as replicates during analysis, corresponding to a total of 2999 different duplicated measurements (a few oligos were spotted multiple times on the arrays). MWG Biotech performed oligonucleotide design, synthesis and spotting. *X. tropicalis *gene sequences were derived from the assembly of public and in-house expressed sequence tags. The temporal expression of *X. tropicalis *during metamorphosis was profiled at 6 time points, using an experimental design consisting of 9 arrays. Each time point was measured three times, with alternating dyes as shown in Figure 1a. This interwoven design was used as a first test set.

From this original design a second test set containing a smaller loop design was derived by picking the combinations of five arrays that connect five time points in a single loop (Figure 1b) and with the first time point as a reference. This results in a balanced loop design

A publicly available spike-in experiment [17] was used as a third test set. This dataset contains 13 spikes-in, or control clones spiked with known concentrations. The control clones were spiked at different concentrations for each of the 7 conditions (Table 7).

The microarray design used for the spike-in experiment was a common reference design, with dye swap for each condition, and the concentrations of spikes ranges from 0 to 10,000 copies per cellular equivalent (cpc), assuming that the total RNA contained 1% poly(A) mRNA and that a cell contained on average 300,000 transcripts. This concentration range covered all biologically relevant transcript levels.

### Probes preparation and microarray hybridization

10 μg of total RNA were used to prepare probes. Labeling was performed with the Invitrogen SuperScript™ Indirect cDNA labeling system (using polyA and random hexamers primers) using the Amersham Cy3 or Cy5 monofunctional reactive dyes. Probe quality was assessed on an agarose minigel and quantified with a Nanodrop ND-1000 spectrophotometer. Dye quantities were equilibrated for hybridization by the amount of fluorescence per ng of cDNA. The arrays were hybridized for 20 h at 45°C according to the manufacturers protocol (QMT ref). Washing was performed in 2× SSC 0.1% SDS at 42°C for 5' and then twice at room temperature in 1× SSC, 0.5× SSC each time for 5'. Arrays were scanned using a GenePix Axon scanner.

### Microarray normalization

The raw intensity data were used for further normalization. No background subtraction was performed. Data were log-transformed and the intensity dependent dye or condition effects were removed by using a local linear fit loess on these log-transformed data (Printtiploess command with default settings as implemented in the limma BioConductor package [18]). As this loess fit not only normalizes the data but also linearizes them, applying it before profile reconstruction is a prerequisite as all linear models used for profile reconstruction assume non linearities to be absent from the data.

For the gene-specific methods (lmbr, lmbr_dye and limmaQual), Loess corrected log-ratios (per print tip) were subjected to an additional quantile normalization step [4,12] as suggested by Vinciotti *et al. *(2005) [3] in order to improve the intercomparability between arrays. It equalizes the distribution of probe intensities for each array in a set of arrays. For the two-stage profile reconstruction methods (anovaFix and anovaMix), corrected log-intensities for the red (*R*_*CORR*_) and green (*G*_*CORR*_) channels were calculated from the Loess corrected log-ratios (*M*_*CORR*_; no additional quantile normalization was done for the two-stage methods) and mean absolute intensities (A) as follows: *R*_*CORR *_= (*A *+ *M*_*CORR*_)/2, and *G*_*CORR *_= (*A *- *M*_*CORR*_)/2.

### Used profile reconstruction methods

Available R implementations (BioConductor [19]) of the presented methods were used to perform the analyses.

#### Gene-specific methods based on log-ratios

Gene-specific profile reconstruction methods apply a linear model on each gene separately. The goal is to estimate the true expression differences between the mRNA of interest and the reference mRNA, from the observed log-ratios. The presented models assume that the expression values have been appropriately pre-processed and normalized [3,20]. The three selected gene-specific models for this study are:

***1) lmbr****, the linear model described by Vinciotti et al. (2005) *[3]:

An observation *y*_*jk *_is the log-ratio of condition *j *and condition *k. *For each gene a vector of *n *observations *y *= (*y*_1_,...,*y*_*n*_) can be represented as

*y *= *Xμ *+ *ε*

where *X *is the design matrix defining the relationship between the values observed in the experiment and a set of independent parameters *μ *= (*μ*_12_, *μ*_13_,...,*μ*_1*T *_representing true expression differences, and *ε *is a vector of errors. The parameters in *μ *are arbitrarily chosen this way for estimation purposes; all expression differences between other conditions *i *and *k *can be calculated from these parameters as *μ*_*ik *_= *μ*_1*k *_- *μ*_1*i*_. The goal is to obtain estimates of the true expression differences $\hat{\mu }$ separately for each gene. Given the assumptions behind the linear model, the least squares estimator for *μ *is [3]

$$\hat{\mu }={\left(X^{t}X\right)}^{-1}X^{t}y$$

***2) lmbr_dye****, an extension of lmbr including a general dye effect:*

The previous model can be extended to include a gene-specific dye effect [3]

*y *= *Xμ *+ *D *+ *ε*

where D is a vector of *n *times a constant value representing the gene-specific dye effect *δ*. Alternatively, one could write *y *= *X*_*D*_*μ*_*D *_+ *ε *where *X*_*D *_is the design matrix *X *with an extra column of ones, and *μ *= (*μ*_12_, *μ*_13_,...,*μ*_1*T*_, *δ*). Note that in the case of dye-balanced designs, the addition of a dye effect will not yield any different estimators for the contrasts of interest. In a balanced design, each column of *X *will have an equal amount of 1's and -1's. I.e. the *i*th column of *X*, corresponding to the true expression difference *μ*_1*i*_, reflects how condition *i *was measured an equal number of times with both dyes. As such, the positive and negative influences of the dye effect will cancel each other out in the estimation of true expression differences. The use of lmbr_dye will thus only render different results compared to lmbr when using it to analyze unbalanced experiments.

In order to estimate all parameters, the matrix *X*_*D *_must be of full rank. If the column representing the dye effect is not linearly independent, the matrix is rank deficient. This situation occurs for example when an array is removed from the loop design used in this paper. In this case, there are an infinite number of possible least squares parameter estimates. Since we expect a single set of parameters, a constraint must be applied (this is done on the dye effect) in which case the true expression estimates are the same as for lmbr.

Lmbr and lmbr_dye were implemented in the R language using the function 'lm' for linear least squares regression.

***3) limmaQual****, the Limma model *[4,20,21] including an array quality adjustment:

The quality adjustment assigns a low weight to poor quality arrays, which can be included in the inference. The approach is based on the empirical reproducibility of the gene expression measures from replicated arrays, and it can be applied to any microarray experiment. The linear model is similar to the model describes by Vinciotti *et al.*, (2005) but the variance of the observations *y *includes the weight term. In this case, the weighted least squares estimator of $\hat{\mu }$ is [20]:

$$\hat{\mu }={\left(X^{t}\sum^{-1}X\right)}^{-1}X^{t}\sum^{-1}y$$

where Σ is the diagonal matrix of weights.

The weights in the limmaQual model are the inverse of estimated array variances, down weighting the observations of lower quality arrays in order to decrease the power to detect differential expression. The method is restricted for use on data from experiments that include at least two degrees of freedom. When testing the array failure in case of the loop design, there is no array level replication for two of the conditions so the array quality weights can not be estimated: the Limma function returns a perfect quality for all the arrays (in this case Σ is a diagonal matrix of 1's).

The fit is by generalized least squares allowing for correlation between duplicate spots or related arrays, implemented in an internal function (*gls.series*) of the Limma package.

#### Two-stage methods based on the log-intensity values

The selected methods correspond to ANOVA (Analysis of variance) models. They can normalize microarray data and provide estimates of gene expression levels that are corrected for potential confounding effects.

Since the global methods are computationally time-consuming, we selected two-stage methods that apply a first stage on all data simultaneously and a second stage on a gene by gene level. These models use partially normalized data as input (i.e., the separate log-intensity values for each channel), as spot effects are explicitly incorporated. They return normalized absolute expression levels for each channel separately (i.e. no ratios), which can then be used to reconstruct the required time profile.

***4) anovaFix****, two-stage ANOVA with fixed effects *[6]:

We denote the loess-normalized log-intensity data by *y*_*ijkgr *_that represents the measurement observed in the array *i*, labeled with the dye *j*, representing the time point *k*, from gene *g *and spot *r*. The first stage is the normalization model:

*y*_*ijkgr *_= *μ *+ *A*_*i *_+ *D*_*j *_+ *AD*_*ij *_+ *r*_*ijkgr*_

where the term *μ *captures the overall mean. The other terms capture the overall effects due to arrays (A), dyes (D) and labelling reactions (AD). This step is called "normalization step" and it accounts for experiment systematic effects that could bias inferences made on the data from the individual genes. The residual of the first stage is the input for the second stage, which models the gene-specific effects:

*r*_*ijkgr *_= *G *+ *SG*_*r *_+ *DG*_*j *_+ *VG*_*k *_+ *ε*_*ijkgr*_

Here G captures the average effect of the gene. The SG effect captures the spot-gene variation and we used it instead of the more global AG array-gene effect. The use of this effect obviates the need for intensity ratios. DG captures specific dye-gene variation and VG (variety-gene) is the effect of interest, the effects due to the time point measured. The MAANOVA fixed model computes least squares estimators for the different effects.

***5) anovaMix****, two-stage ANOVA with mixed effects *[6,16]:

The model applied is exactly the same as anovaFix, but in this case the SG effect was treated as a random variable, meaning that if the experiment were to be repeated, the random spot effects would not be exactly reproduced, but they would be drawn from a hypothetical population. A mixed model, where some variables are treated as random, allows for including multiple sources of variation.

We used the default method to solve the mixed model equation, the REML (restricted maximum likelihood) method. Duplicated spots were treated as independent measurements of the same gene. For MAANOVA and Limma packages the option to do so is available, for lmbr and lmbr_dye duplicated spots were taken into account by the design matrix.

### Profile reconstruction

Applying the gene-specific methods mentioned above results in estimated differences in log-expression between a test and a reference condition or in log-ratios. To reconstruct from the different designs a time profile, the first time point was chosen as the reference. A gene-specific reconstructed profile thus consists of a vector which contains as entries ratios of the measured expression level of that gene at each time point except the first, relative to its expression value at the first time point. For instance, for the loop design shown in Table 1 the profile contains 4 ratios.

In contrast to the gene-specific methods, two-stage methods estimate the absolute gene expression level for each time point rather than log-ratios. In this case, for the loop design shown in Table 1, the profile contains 5 gene expression levels.

### Comparison of profile reconstruction

To assess the influence of using different methodologies on the profile reconstruction, the following similarity measures were used to compare the consistency in reconstructing profiles for the same gene between the compared methods:

1. Overall similarity in the estimated ratios: we assessed the similarity between the estimations of each single ratio of the time profile generated by two methods using the Pearson correlation. Since two-stage methods estimate gene expression levels (variety-gene effect in the model) instead of log-ratios, we converted these absolute values into log-ratios by subtracting from the absolute expression levels estimated for each of the conditions the estimated level of the first time point (the reference).

2. Profile shape similarity: the profile shape reflects the expression behaviour of a gene over time. For each single gene, we computed the mutual similarity between profile estimates obtained by any combination of two methods. To make profiles consisting of log-ratios obtained by the gene-specific methods comparable with the profiles estimated by the two-stage methods, we extended the log-ratios profile by adding as a first time point a zero. This represents the contrast between the expression value of the first time point against itself in log-scale (Figure 2).

### Profile Similarity

The mutual similarity was computed as the cosine similarity, which corresponds to the angle between two vectors representing genes *i *and *j *with profiles *P*_*i *_and *P*_*j*_.

$$\cos(P_{i},P_{j})=\frac{P_{i}\cdot P_{j}}{\left\|Pi\right\|\left\|Pj\right\|}$$

All profiles were mean centered, i.e. data have been shifted by the mean of the profile ratios to have an average of zero for each gene, prior to computing the cosine similarity. With centered data, the cosine similarity can also be viewed as the correlation coefficient, and it ranges between -1 (opposite shape) and 1 (similar shape), 0 being no correlation. The cosine similarity only considers the angle between the vectors focusing on the shape of the profile. As a result, it ignores the magnitude of ratios of the profiles, resulting in relatively high similarities for false positives (i.e. "flat profiles", genes that do not change their expression profile over time, but for which the noise profile corresponds by chance to other gene profiles).

No variance normalization was performed on the profiles to preserve their shape. Instead of normalizing by the variance, the profiles were filtered using the standard deviation.

### Filtering flat profiles

Constitutively expressed genes or genes for which the expression did not significantly change over the conditions were filtered by removing genes of which the variance in expression over different conditions was lower than a fixed threshold (the following range of thresholds was tested: 0.1, 0.2, 0.4). A pair wise similarity comparison was made for all profile estimates (corresponding to the same gene) that were above the filtering threshold in at least one of the two methods compared. Similar results were obtained when applying as a filter that all profile estimates had to be above the filtering threshold in both methods compared (data not shown).

## Authors' contributions

AF performed the analysis and wrote the manuscript. RT and NP were responsible for the microarray hybridization. NP and GB critically read the draft. KE contributed to the analysis. KM coordinated the work and revised the manuscript. All authors read and approved the final manuscript.

## Supplementary Material

Additional file 1 Plot of corresponding ratios estimated by two linear methods. Comparison of corresponding ratios estimated by lmbr and anovaMix using the loop design. The line indicates the identity between both methods and most of the points are situated near this identity line. Click here for file

Additional file 2 Pairwise correlation between ratios estimated for the interwoven design. The table shows the pairwise correlation between ratios estimated by each pair of methods (columns 1 and 2) for the interwoven design. The ratios correspond to the change in expression compared to the first time point. The last column corresponds to the mean correlation of the 5 estimations. Click here for file

Additional file 3 Mean similarity between profiles using different filtering thresholds. Values in the table correspond to the similarity between any two methods, expressed as the mean profile similarity of the genes. Results are shown for both the interwoven and loop design using different filtering thresholds. Since the loop design is balanced with respect to the dyes, the results for lmbr and lmbr_dye were the same (see 'Methods' section), which is why they are not treated differently. A) No filtering applied, similarity is assessed for all 2999 profile estimates, B) a filtering threshold (SD) is used on all profiles estimated by each of the methods, a pairwise similarity comparison is made for all profile pairs (corresponding to the same gene) estimated by each of the two methods compared, for which at least one profile is above the filtering threshold (SD >0.1, 0.2, 0.4 respectively). Click here for file

Additional file 4 Effect of array failure for the interwoven design. The table shows the effect of array failure in reconstructing profiles from an interwoven design. Profile similarities were assessed using the cosine similarity. The different methods for which the influence of the failure was assessed are represented in the columns. Each row shows the mean cosine similarity between the corresponding profiles estimated from the complete design and those obtained from a defect design (where one array was removed compared to the complete design). Mean: shows the overall mean similarity for a given method. Click here for file

Additional file 5 Effect of array failure for the loop design. The table shows the effect of array failure in reconstructing profiles from a loop design. Profile similarities were assessed using the cosine similarity. The different methods for which the influence of the failure was assessed are represented in the columns. Each row shows the mean cosine similarity between the corresponding profiles estimated from the complete design and those obtained from a defect design (where one array was removed compared to the complete design). Mean: shows the overall mean similarity for a given method. Click here for file
