# Supplementary material for: Evaluation of time profile reconstruction from complex two-color microarray designs
Source: BMC Bioinformatics. 2008 Jan 3;9:1. doi: 10.1186/1471-2105-9-1 (PMC2265676; doi:10.1186/1471-2105-9-1)

Figure S1: Comparison of corresponding ratios estimated by two linear methods (lmbr and anovaMix) using the loop design. The line indicates the identity between both methods and most of the points are situated near this identity line.

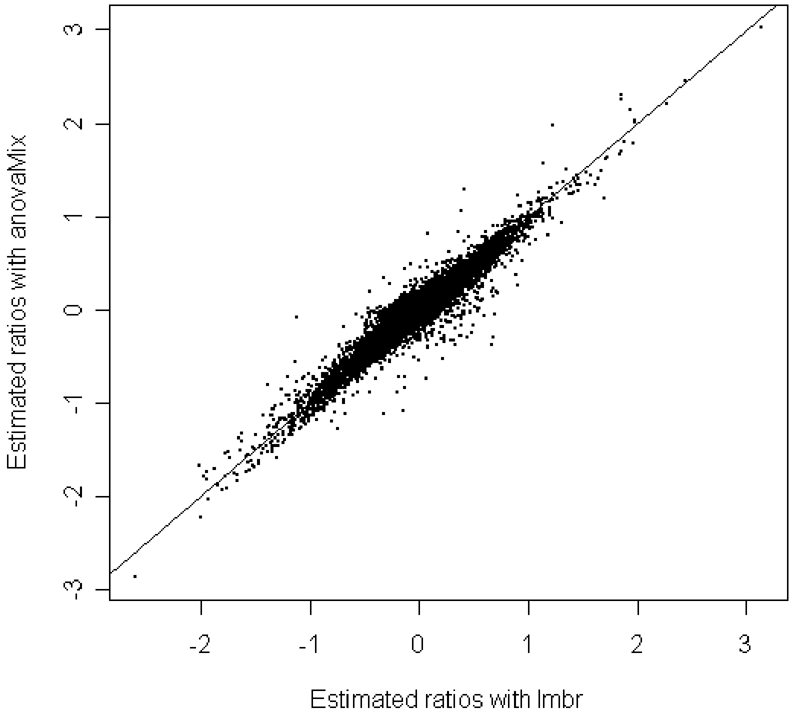

Supplement: Additional file 1 — Plot of corresponding ratios estimated by two linear methods. Comparison of corresponding ratios estimated by lmbr and anovaMix using the loop design. The line indicates the identity between both methods and most of the points are situated near this identity line. [file 1471-2105-9-1-S1.pdf]
