# Supplementary material for: Evaluation of time profile reconstruction from complex two-color microarray designs
Source: BMC Bioinformatics. 2008 Jan 3;9:1. doi: 10.1186/1471-2105-9-1 (PMC2265676; doi:10.1186/1471-2105-9-1)
Supplement: Additional file 2 — Pairwise correlation between ratios estimated for the interwoven design. The table shows the pairwise correlation between ratios estimated by each pair of methods (columns 1 and 2) for the interwoven design. The ratios correspond to the change in expression compared to the first time point. The last column corresponds to the mean correlation of the 5 estimations. [file 1471-2105-9-1-S2.pdf]

Table S1: Pairwise correlation between ratios estimated by each pair of methods (columns 1 and 2) for the interwoven design. The ratios correspond to the change in expression compared to the first time point. The last column corresponds to the mean correlation of the 5 estimations.

Method1	Method2	T2/T1	T3/T1	T4/T1	T5/T1	T6/T1	mean
lmbr	lmbr_dye	0.9983	0.9963	0.9536	0.9694	0.9485	0.9732
lmbr	limmaQual	0.9991	0.9997	0.9991	0.9962	0.9991	0.9986
lmbr	anovaFix	0.9798	0.9871	0.9483	0.9645	0.9515	0.9662
lmbr	anovaMix	0.9605	0.9805	0.9353	0.9509	0.9426	0.9540
lmbr_dye	limmaQual	0.9976	0.9941	0.9451	0.9862	0.9352	0.9716
lmbr_dye	anovaFix	0.9823	0.9893	0.9910	0.9745	0.9862	0.9847
lmbr_dye	anovaMix	0.9621	0.9831	0.9834	0.9627	0.9800	0.9742
limmaQual	anovaFix	0.9774	0.9851	0.9391	0.9733	0.9402	0.9630
limmaQual	anovaMix	0.9568	0.9783	0.9256	0.9597	0.9311	0.9503
anovaFix	anovaMix	0.9779	0.9946	0.9930	0.9906	0.9943	0.9901
