# Supplementary material for: Evaluation of time profile reconstruction from complex two-color microarray designs
Source: BMC Bioinformatics. 2008 Jan 3;9:1. doi: 10.1186/1471-2105-9-1 (PMC2265676; doi:10.1186/1471-2105-9-1)
Supplement: Additional file 3 — Mean similarity between profiles using different filtering thresholds. Values in the table correspond to the similarity between any two methods, expressed as the mean profile similarity of the genes. Results are shown for both the interwoven and loop design using different filtering thresholds. Since the loop design is balanced with respect to the dyes, the results for lmbr and lmbr_dye were the same (see 'Methods' section), which is why they are not treated differently. A) No filtering applied, similarity is assessed for all 2999 profile estimates, B) a filtering threshold (SD) is used on all profiles estimated by each of the methods, a pairwise similarity comparison is made for all profile pairs (corresponding to the same gene) estimated by each of the two methods compared, for which at least one profile is above the filtering threshold (SD >0.1, 0.2, 0.4 respectively). [file 1471-2105-9-1-S3.pdf]

Table 2S - Mean similarity between profiles for both the interwoven and loop design using different filtering thresholds

Values in the table correspond to the similarity between any two methods, expressed as the mean profile similarity of the genes. Since the loop design is balanced with respect to the dyes, the results for lmbr and lmbr_dye were the same (see 'Methods' section), which is why they are not treated differently.
A) No filtering applied, similarity is assessed for all 2999 profile estimates, B) a filtering threshold (SD) is used on all profiles estimated by each of the methods, a pairwise similarity comparison is made for all cognate profile pairs estimated by each of the two methods compared, for which at least one profile is above the filtering threshold (SD >0.1, 0.2, 0.4 respectively).

A)

	Interwoven design				Loop design		
	lmbr	lmbr_dye	limmaQual	anovaFix	lmbr/ lmbr_dye	limmaQual	anovaFix
lmbr_dye	0.9477				1.0000		
limmaQual	0.9940	0.9359			0.9844		
anovaFix	0.9321	0.9572	0.9157		0.9514	0.9252	
anovaMix	0.9138	0.9373	0.8989	0.9767	0.9186	0.8934	0.9611

B)

SD > 0.1

	Interwoven design				Loop design		
	lmbr	lmbr_dye	limmaQual	anovaFix	lmbr/ lmbr_dye	limmaQual	anovaFix
lmbr_dye	0.9575				1.0000		
limmaQual	0.995	0. 9472			0.9885		
anovaFix	0. 9462	0. 9708	0. 933		0.9681	0.9482	
anovaMix	0. 9328	0. 9558	0. 9205	0. 9824	0.9418	0.9226	0.9706

SD > 0.2

	Interwoven design				Loop design		
	lmbr	lmbr_dye	limmaQual	anovaFix	lmbr/ lmbr_dye	limmaQual	anovaFix
lmbr_dye	0. 9732				1.0000		
limmaQual	0. 9972	0. 9669			0.9919		
anovaFix	0. 9689	0. 989	0. 9616		0.9856	0.9725	
anovaMix	0. 9613	0. 9798	0. 9545	0. 99	0.9629	0.949	0.9776

SD > 0.4

	Interwoven design				Loop design		
	lmbr	lmbr_dye	limmaQual	anovaFix	lmbr/ lmbr_dye	limmaQual	anovaFix
lmbr_dye	0. 9834				1.0000		
limmaQual	0. 998	0. 9799			0.992		
anovaFix	0. 98	0. 9948	0. 9755		0.9961	0.9851	
anovaMix	0. 9748	0. 9905	0. 9701	0. 9959	0.9866	0.9728	0.9905
