# Supplementary material for: Evaluation of time profile reconstruction from complex two-color microarray designs
Source: BMC Bioinformatics. 2008 Jan 3;9:1. doi: 10.1186/1471-2105-9-1 (PMC2265676; doi:10.1186/1471-2105-9-1)
Supplement: Additional file 4 — Effect of array failure for the interwoven design. The table shows the effect of array failure in reconstructing profiles from an interwoven design. Profile similarities were assessed using the cosine similarity. The different methods for which the influence of the failure was assessed are represented in the columns. Each row shows the mean cosine similarity between the corresponding profiles estimated from the complete design and those obtained from a defect design (where one array was removed compared to the complete design). Mean: shows the overall mean similarity for a given method. [file 1471-2105-9-1-S4.pdf]

Table S3: Assessing the effect of array failure in reconstructing profiles from an interwoven design. Profile similarities were assessed using the cosine similarity. The different methods for which the influence of the failure was assessed are represented in the columns. Each row shows the mean cosine similarity between the corresponding profiles estimated from the complete design and those obtained from a defect design (where one array was removed compared to the complete design). Mean: shows the overall mean similarity for a given method.

Number of Array removed	lmbr	lmbr_dye	limmaQual	anovaFix	anovaMix	Conditions affected
1	0.9292	0.9443	0.9150	0.9598	0.9545	T2/T3
2	0.9252	0.9660	0.8953	0.9390	0.9484	T1/T3
3	0.8778	0.7200	0.8629	0.8026	0.7816	T3/T5
4	0.9649	0.9727	0.9442	0.9569	0.9588	T4/T1
5	0.9762	0.9818	0.9581	0.9605	0.9615	T6/T1
6	0.9518	0.9648	0.9475	0.9662	0.9681	T5/T2
7	0.8929	0.8638	0.9126	0.8759	0.9001	T5/T6
8	0.9275	0.9367	0.9336	0.9438	0.9525	T2/T4
9	0.9240	0.9428	0.9071	0.9536	0.9559	T4/T6
Mean	0.9299	0.9215	0.9196	0.9287	0.9313	
