# Supplementary material for: Evaluation of time profile reconstruction from complex two-color microarray designs
Source: BMC Bioinformatics. 2008 Jan 3;9:1. doi: 10.1186/1471-2105-9-1 (PMC2265676; doi:10.1186/1471-2105-9-1)
Supplement: Additional file 5 — Effect of array failure for the loop design. The table shows the effect of array failure in reconstructing profiles from a loop design. Profile similarities were assessed using the cosine similarity. The different methods for which the influence of the failure was assessed are represented in the columns. Each row shows the mean cosine similarity between the corresponding profiles estimated from the complete design and those obtained from a defect design (where one array was removed compared to the complete design). Mean: shows the overall mean similarity for a given method. [file 1471-2105-9-1-S5.pdf]

Table S4: Assessing the effect of array failure in reconstructing profiles from a loop design. Profile similarities were assessed using the cosine similarity. The different methods for which the influence of the failure was assessed are represented in the columns. Each row shows the mean cosine similarity between the corresponding profiles estimated from the complete design and those obtained from a defect design (where one array was removed compared to the complete design). Mean: shows the overall mean similarity for a given method.

Array removed	lmbr	lmbr_dye	limmaQual	anovaFix	anovaMix	Conditions affected
1	0.2381	-	-	0.6818	0.6163	T1/T3
2	0.2481	-	-	0.6318	0.5444	T3/T5
3	0.2548	-	-	0.7075	0.6288	T5/T6
4	0.2610	-	-	0.7012	0.6102	T6/T4
5	0.2369	-	-	0.6652	0.5937	T4/T1
Mean	0.2478	-	-	0.6775	0.5987	
